# Effects of beetroot juice supplementation on intermittent high-intensity exercise efforts

**DOI:** 10.1186/s12970-017-0204-9

**Published:** 2018-01-05

**Authors:** Raúl Domínguez, José Luis Maté-Muñoz, Eduardo Cuenca, Pablo García-Fernández, Fernando Mata-Ordoñez, María Carmen Lozano-Estevan, Pablo Veiga-Herreros, Sandro Fernandes da Silva, Manuel Vicente Garnacho-Castaño

**Affiliations:** 1 Physical Activity and Sport Sciences, College of Health Sciences, Alfonso X El Sabio University, Madrid, Spain; 2 TecnoCampus. GRI-AFIRS, School of Health Sciences, Pompeu Fabra University, Mataró, Barcelona, Spain; 3 NutriScience, C/Paco León, 1, 14010 Córdoba, Spain; 4 Physical Activity and Sport Sciences, Physical Education Departament, University of Lavras, Lavras, Brazil

**Keywords:** Beet, Ergogenic aids, Exercise, Sport supplement

## Abstract

Beetroot juice contains high levels of inorganic nitrate (NO_3_^−^) and its intake has proved effective at increasing blood nitric oxide (NO) concentrations. Given the effects of NO in promoting vasodilation and blood flow with beneficial impacts on muscle contraction, several studies have detected an ergogenic effect of beetroot juice supplementation on exercise efforts with high oxidative energy metabolism demands. However, only a scarce yet growing number of investigations have sought to assess the effects of this supplement on performance at high-intensity exercise. Here we review the few studies that have addressed this issue. The databases Dialnet, Elsevier, Medline, Pubmed and Web of Science were searched for articles in English, Portuguese and Spanish published from 2010 to March 31 to 2017 using the keywords: beet or beetroot or nitrate or nitrite and supplement or supplementation or nutrition or “sport nutrition” and exercise or sport or “physical activity” or effort or athlete. Nine articles fulfilling the inclusion criteria were identified. Results indicate that beetroot juice given as a single dose or over a few days may improve performance at intermittent, high-intensity efforts with short rest periods. The improvements observed were attributed to faster phosphocreatine resynthesis which could delay its depletion during repetitive exercise efforts. In addition, beetroot juice supplementation could improve muscle power output via a mechanism involving a faster muscle shortening velocity. The findings of some studies also suggested improved indicators of muscular fatigue, though the mechanism involved in this effect remains unclear.

## Background

Because of the increase in competitive equality in high level sport, a 0.6% performance improvement is today considered sufficient to make a difference [1]. In this setting of high competition, athletes often look to nutritional supplements to boost their performance [2]. However, most statements about the potential effects on sport performance or health that appear on the labels of many products are not backed by clear scientific evidence [2]. Because of this, institutions such as the Australian Institute of Sport (AIS) have created a system to classify supplements according to their effects on performance based on confirmed scientific evidence [3]. Thus, dietary supplements assigned to class A have been proven with a high level of evidence to improve exercise performance in certain modalities when taken in appropriate amounts. The only substances in this class are β-alanine, sodium bicarbonate, caffeine, creatine and beetroot juice [4]. However, it is thought that the effect of a given supplement on performance besides the recommended dose may be specific to each sport’s modality [5]. This, in turn, will depend on the energy and/or mechanical requirements of each form of exercise such that some supplements will have an ergogenic effect on some types of exercise efforts and have no effects on other types.

The relationship between exercise intensity and time to exhaustion is hyperbolic [6] as it is directly linked to the prevailing energy producing systems during exercise [7]. Thus, depending on their bioenergetics, the different exercise efforts can be classified according to exercise duration. This means we can differentiate between explosive efforts, high-intensity efforts and endurance-intensive efforts [8]. Explosive efforts are those lasting under 6 s in which the main energy metabolism pathway is the high-energy phosphagen system and there is some participation also of glycolysis [9, 10], which gradually contributes more energy until 50% at 6 s [9]. High-intensity efforts are those of duration longer than 6 s and shorter than 1 min [11]. These efforts are characterized by a major contribution of glycolytic metabolism and smaller contribution of high-energy phosphagens and oxidative phosphorylation [8]. Finally, intensive endurance efforts are those lasting longer than 60 s and whose main energy producing system is oxidative phosphorylation [8].

Beetroot juice is used as a supplement because it may serve as a precursor of nitric oxide (NO) [12]. The mechanism of NO synthesis is thought to be via the catabolism of arginine by the enzyme NO synthase [13]. Effectively, arginine supplementation has been shown to increase NO levels [14]. An alternative mechanism of NO genesis is mediated by inorganic nitrate (NO_3_^−^). This means that the high amounts of NO_3_^−^ present in beetroot juice are able to increase NO levels in the organism.

**Fig. 1:**
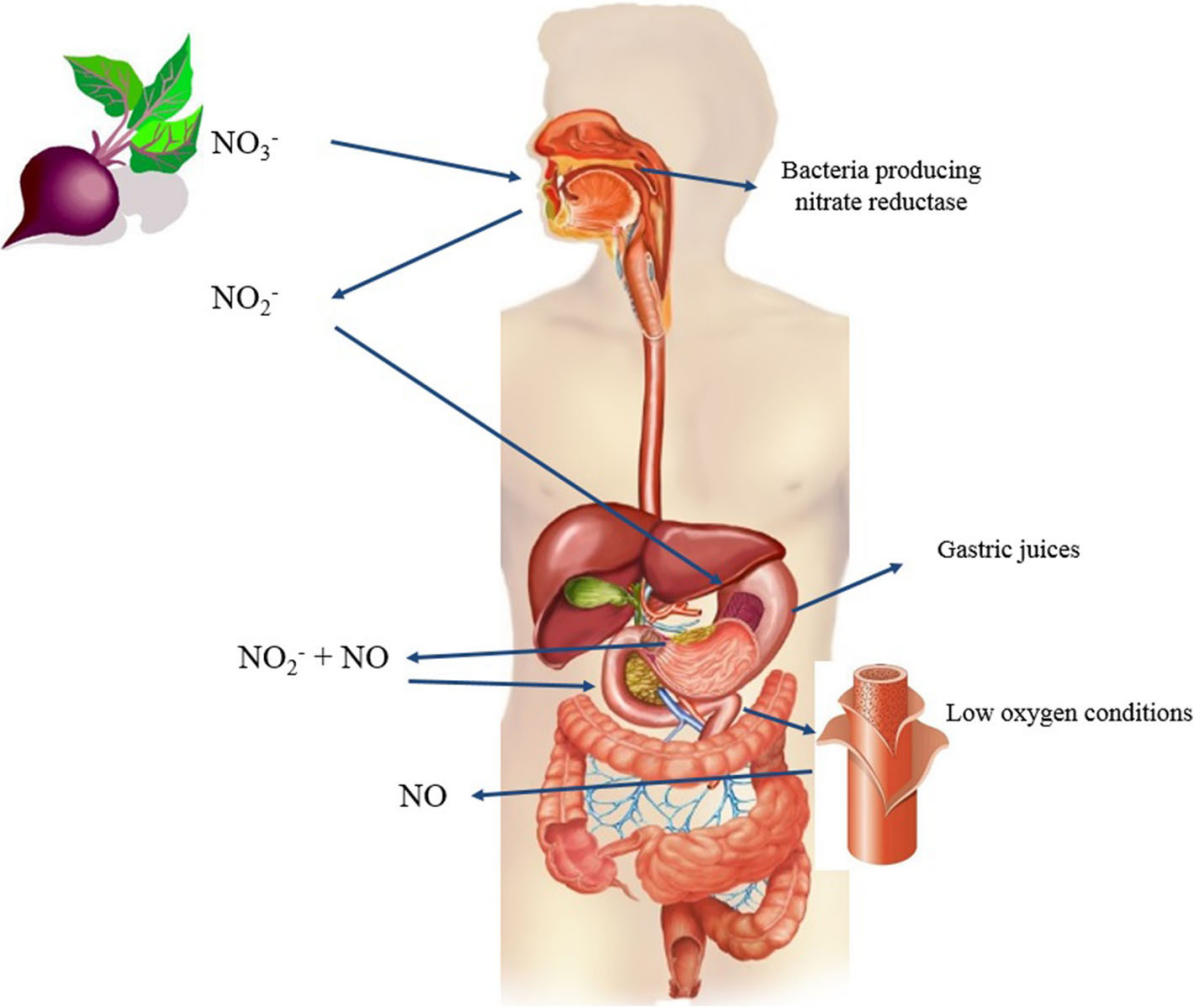
Conversion of NO_3_^−^ in beetroot juice to NO. The diagram shows how ingested NO_3_^−^ is transformed by bacteria in the mouth containing nitrite reductase to NO_2_^−^. Once in the gut, NO_2_^−^ enters the bloodstream and, under conditions of hypoxia, is used to generate NO

In the mouth, some 25% of dietary NO_3_^−^ is reduced by NO_3_^−^ reductase produced by microorganisms [15] to nitrite (NO_2_^−^) [16]. This NO_2_^−^ is then partially reduced to NO through the actions of stomach acids which is later absorbed in the gut [17]. Some of this NO_2_^−^ enters the bloodstream, and, in conditions of low oxygen levels, will be converted into NO [18] (Fig. 1).

Nitrous oxide has numerous physiological functions including haemodynamic and metabolic actions [19, 20]. Mediated by guanylyl cyclase [21], NO has an effect on smooth muscle fibres causing blood vessel dilation [22]. This vasodilation effect increases blood flow to muscle fibres [23] promoting gas exchange [24]. NO also induces gene expression [25], enhancing biogenesis [26] and mitochondrial efficiency [27]. All these effects can favour an oxidative energy metabolism. In effect, though not all [28–31], numerous investigations have noted that beetroot juice supplementation boosts performance in exercise modalities involving intensive endurance efforts in which the dominant type of energy metabolism is oxidative [24, 27, 32–45].

To date, several reviews of the literature have assessed the effects of beetroot juice supplements on physical exercise [12, 46–49]. In addition, given that NO can potentiate the factors that limit performance when executing actions in which the predominant metabolism is oxidative, two recent reviews have explored the positive effects of this form of supplementation on endurance exercise [50, 51]. Thus, the different studies showed that beetroot juice supplementation was effective at: lowering VO_2_ by −6% during a swimming test conducted at an intensity equivalent to the ventilatory threshold (VT) [27]; lowering VO_2_ by −3% during a kayaking test conducted at 60% VO_2max_ [38] and during a cycle ergometry test conducted by recreation sport athletes [45] and cyclists [34] at 45–70% VO_2max_; increasing performance by 12–17% in cycle ergometry tests until exhaustion conducted at intensities of 60 to 90% VO_2max_ by recreation sport athletes [37, 42], and by 22% when conducted at a 70% intensity between VT and VO_2max_ [36]; and finally, improving times by 2.8% in trained cyclists conducting cycle ergometery tests of 4 km [33], 10 km (1.2%) [34], 16 km (2.7%) [33] and 50 miles (0.8%) [35]. However, besides the effects of NO mentioned above, other impacts need to be considered. Accordingly, it has been described that the effect of increased blood flow induced by NO is specific to type II muscle fibres [20]. Moreover, in type II muscle fibres, beetroot juice intake has been found to improve the release and later reuptake of calcium from the sarcoplasmic reticulum [52]. This could translate to an increased capacity for muscle strength production of these type II muscle fibres. Such effects of NO could mean a physiological advantage for efforts involving the recruitment of type II muscle fibres, such as intermittent, high-intensity efforts. Hence, given the scarce yet growing number of studies that have addressed the effects of beetroot juice supplementation on this type of intermittent, high-intensity effort [38, 53–60], here we review the results of experimental studies that have specifically examined in adults (whether athletes or not) the effects of beetroot juice supplementation on intermittent, high-intensity efforts.

## Methodology

We identified all studies that have assessed the effects of BJ supplementation on intermittent, high-intensity efforts by searching the databases Dialnet, Elsevier, Medline, Pubmed and Web of Science published up until March 31, 2017 using the keywords: beet OR beetroot OR nitrate OR nitrite (concept 1) AND supplement OR supplementation OR nutrition OR “sport nutrition” (concept 2) AND exercise OR sport OR “physical activity” OR effort OR athlete (concept 3).

Two of the present authors (E.C and P.G-F) first eliminated duplicate articles and then removed descriptions of studies that were not experimental, were not written in English or Spanish, or were published before 2010. This meant that all the studies reviewed were published over the period January 1, 2010 to March 31, 2017. Next, these two same authors applied a set of exclusion criteria to ensure the selection only of studies specifically designed to assess the effects of BJ supplementation on intermittent, high-intensity efforts:

- Studies performed in non-adults (samples including subjects aged <18 or >65 years).
- Studies conducted in vitro or in animals.
- Studies in which the direct effects of BJ were not determined.
- Studies in which impacts were examined on exercises that did not comply with the characteristics of intermittent, high-intensity efforts.

If there was disagreement about whether a given study met the inclusion/exclusion criteria, the opinion of a third researcher (F.M-O) was sought.

## Results

### Study selection

**Fig. 2:**
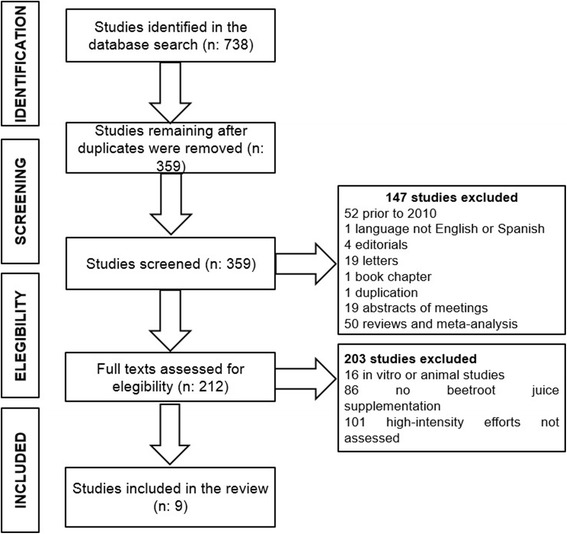
Article selection

Of 738 studies identified in the search, 359 were left after eliminating repeated records. Once, the titles and abstract of these 359 publications were reviewed, 212 full text articles were indentified and retrieved for assessment, of which 9 articles met the elegibility criteria (Fig. 2).

### Study characteristics

The nine studies selected for our review included a total of 120 subjects, 107 of whom were men and 13 women.

In five of these studies [38, 53, 54, 57, 59], the effects of a single beetroot juice supplement (acute effects) were assessed. The supplement was taken 120 min before exercise in one study [53], 150 min before exercise in two [57, 59] and 180 min before exercise in the remaining two [38, 54].

In the remaining four studies, the effects of chronic beetroot juice supplementation were examined [55, 56, 58, 60]. The supplementation periods were 5 days in one study [60], 6 days in two [55, 58] and 7 days in the fourth study [56].

Doses of NO_3_^−^ ingested ranged from ~5 mmol [38] to ~11.4 mmol [57]. In addition, one study examined the efficacy of beetroot juice taken separately or in combination with sodium phosphate [55].

In four of the nine studies reviewed, participants were competition athletes [38, 55, 57, 59] and in the other five they were recreation sport or low-level competition athletes [53, 54, 56, 58, 60]. Only one of the study populations included athletes of individual sports modalities [38], the rest of the studies were conducted in players of team sports [53–60].

The tests used to assess performance were a 30-s duration cycle ergometer test in one [59] and high-intensity, intermittent exercises in the remaining studies with work intervals ranging from 6 s [58] to 60 s [60] and rest periods from 14 s [56] to 4 min [60]. The types of tests employed were running at maximum speed in three studies [55–57], cycle ergometry in four [53, 54, 59, 60], one of which was an isokinetic test [59], a kayak ergometer test in one [38] and bench press strength training in the remaining study [58].

The beetroot juice intervention led to significantly improved performance in four of the studies [54, 56, 58, 60], while in another four no such effects were observed [38, 55, 57, 59]. In the remaining study, an ergolytic, or reduced performance, effect was noted in relation to the placebo treatment.

### Study results

In Table 1 we summarize the results of the nine studies reviewed and provide details on the participants, experimental conditions, supplement regimens, and performance tests employed.

**Table 1:** Summary of the results obtained in studies examining the impacts of beetroot juice supplements on intermittent high intensity exercise performance

Reference	Subjects	Study design	Dose	Exercise test	Results
Muggeridge et al. [38]	Trained kayakers (male, *n* = 8) (VO_2peak_ 49.0 ± 6.1 ml·kg·min^−1^)	Single-blind, randomized, cross-over	5 mmol NO_3_^−^ (180 min before)	Kayak ergometer: 5 × 10 s sprint-rest 50 s	+4% average power (420 ± 23 vs 404 ± 24 W)
Martin et al. [53]	Recreation team sport players (male,*n* = 16) (VO_2peak_ 47.2 ± 8.5 ml·kg·min^−1^)	Double-blind, randomized, cross-over	6.4 mmol NO_3_^−^ (120 min before)	Cycle ergometer: sets until exhaustion of 8 s– rest 30 s	−13% reps (13 ± 5 vs 15 ± 6) and −17% total work (49.2 ± 24.2 vs 57.8 ± 34.0 kJ)
Aucouturier et al. [54]	Recreation team sport players (male, *n* = 12) (VO_2peak_ 46.6 ± 3.4 ml·kg·min^−1^)	Single-blind, randomized, cross-over	10.9 mmol NO_3_^−^ (180 min before)	Cycle ergometer: sets until exhaustion of 15 s at 170% MAP–rest 30 s	+20% reps^*^ (26.1 ± 10.7 vs 21.8 ± 8.0) and 18% total workload^*^ (168.2 ± 60.2 vs 142.0 ± 46.8 kJ)
Buck et al. [55]	Amateur team sport players (female, *n* = 13) (VO_2peak_ not specified)	Double-blind, randomized, Latin-square	BJ: 6.4 mmol NO_3_^−^ (6 days) BJ + SP: 6.4 mmol NO_3_^−^ + 50 mg·kg lean mass SP (6 days)	PRE, MID and POST simulation team sport matches: 6×(20 m sprint + rest 25 s)	BJ: −0.2% total sprint time per set (69.8 ± 4.9 vs 69.97 ± 4.2) BJ + SP: −2% total sprint time per set (68.9 ± 5.1 vs 69.97 ± 4.2)
Thompson et al. [56]	Recreation team sport players (male, *n* = 16) (VO_2peak_ 50 ± 7 ml·kg·min^−1^)	Double-blind, randomized, cross-over	12.8 mmol NO_3_^−^ (7 days)	MID and POST simulated team-sport matches: 2×[5×(6 s cycle ergometry sprint + rest 14 s)]	5% work volume at MID^*^ (63 ± 20 vs 60 ± 18 kJ), 2% POST (60 ± 17 vs 59 ± 16 kJ) and 4% whole session^*^ (123 ± 19 vs 119 ± 17 kJ)
Clifford et al. [57]	Competition team sport players (male, *n* = 20) (VO_2peak_ not specified)	Double-blind, independent groups design	11.4 mmol NO_3_^−^ (150 min before)	2xRST: 20×(30 m sprint–rest 30 s)	-1% average sprint time RST1 (4.65 ± 0.3 vs 4.7 ± 0.2 s) and −2% RST2 (4.66 ± 0.2 vs 4.77 ± 0.2 s) and −2% fastest sprint RST1 (4.41 ± 0.2 vs 4.48 ± 0.1 s) and −3%RST2 (4.38 ± 0.2 vs 4.53 ± 0.2 s)
Mosher et al. [58]	Recreation sport players (male, *n* = 12) (VO_2peak_ not specified)	Double-blind, randomized, cross-over	6.4 mmol NO_3_^−^ (6 days)	Bench press: 3×(maximum number reps at 60% 1 RM)	+ 19% weight lifted in session and improved no. of reps S1^*,^ S2^*,^ S3^*^ and whole session. ^*^improvements not specified
Rimer et al. [59]	Competition sport players (male, *n* = 13) (VO_2peak_ not specified)	Double-blind, randomized, cross-over	11.2 mmol NO_3_^−^ (150 min before)	Isokinetic cycle ergometer: Wingate 30-s test	-1% peak power (1173 ± 255 vs 1185 ± 249 W) and −1% total work (22.8 ± 4.8 vs 23 ± 4.8 W)
Wylie et al. [60]	Recreation team sport players (male, *n* = 10) (VO_2peak_ 58 ± 8 ml·kg·min^−1^)	Double-blind, randomized, cross-over design	8.4 mmol NO_3_^−^ (5 days)	Cycle ergometer: 24 x (6 s sprint–rest 24 s) Cycle ergometer: 7 x (30 s sprint–rest 4 min) Cycle ergometer: 6 x (60 s sprint–rest 60 s)	+5% mean average power^*^ (568 ± 136 vs 539 ± 136 W) and +1% mean peak power (792 ± 159 vs 782 ± 154 W) in 24 x (6 s sprint–rest 24 s);−1% mean average power (558 ± 95 vs 562 ± 94 W) and −1% mean peak power (768 ± 157 vs 776 ± 142 W) in 7 x (30 s sprint–rest 4 min)

*BJ* Beetroot juice, *MID* Half-time simulation match, *n* Sample size; *no* Number, *NO*_*3*_^*−*^ nitrate concentration in the drink, *MAP* Maximum aerobic power, *POST* End simulation match, *PRE* Before simulation match, *Rep* Repetition, *RST* Repeated sprint test, *SP* Sodium phosphate, *VO*_*2peak*_ Peak oxygen consumption, ^*^ statistically significant differences

## Discussion

### Effects of chronic supplementation with beetroot juice on intermittent, high-intensity exercise efforts

Four of the studies reviewed tested the effects of taking beetroot juice supplements for 5 to 7 days on intermittent, high-intensity efforts [55, 56, 60] or on a resistance training session [58]. Three of these studies detected a significant effect of beetroot juice supplementation [56, 58, 60] while in the remaining study, no significant difference compared with the placebo was noted [55].

#### Effects of chronic supplementation with beetroot juice on resistance training

Resistance training is used to improve muscular hypertrophy, strength, power and muscular endurance [61]. Training sessions targeting muscle hypertrophy include workloads of around 70–85% 1 RM and 8–12 repetitions, while those aiming to improve muscular endurance include loads of around 50% 1 RM and some 15–25 repetitions [62]. Such exercise sessions are largely dependent on glycolytic metabolism; the lactate threshold in resistance training exercises such as half squat is detected at ~25% 1 RM [63, 64]. To determine the effects of 6 days of beetroot juice supplementation (6.4 mmol NO_3_) on resistance training sessions designed to improve local muscular hypertrophy and endurance, in the study by Mosher et al. reviewed here [58], the number of bench press repetitions accomplished in three sets using loads equivalent to 60% 1 RM was recorded. Results indicated that supplementation increased the number of repetitions in the three exercise sets improving session performance by 18.9%.

In an earlier investigation, the effects of sodium bicarbonate supplements were assessed in a similar study to the one by Mosher et al. [58]. Subjects performed 3 sets until exhaustion with loads of 10–12 RM in three exercises targeting the lower limbs [65]. Results indicated that, like the beetroot juice, sodium bicarbonate supplementation led to more repetitions in the session [65]. However, in parallel with the increasing number of repetitions, blood lactate concentrations also rose (~2.5 mmol) [65]. This was not observed in Mosher’s study [58].

If we consider the nature of resistance training, the athlete passes from a resting condition to a situation demanding high energy levels during the first repetitions of a set. Because the phosphagen system is the main energy pathway in rest-exercise transitions [66], phosphocreatine reserves may be depleted in response to a resistance training exercise set. Recovering these reserves takes some 3–5 min [67]. Given that phosphocreatine resynthesis is dependent on oxidative metabolism [68] and that beetroot juice has an ergogenic effect on exercise modalities with a major oxidative metabolism component [50], it could be that this supplement accelerated this recovery during the rest period in Mosher’s study (2 min) and thus avoided progressive phosphocreatine depletion throughout the session. In turn, this faster rate of resynthesis would attenuate the increasing levels of adenosine diphosphate (ADP) and inorganic phosphates [68]. Both these metabolites have been associated with the appearance of muscular fatigue [69]. Hence, by delaying the build-up of critical levels of these metabolites, the appearance of fatigue will be delayed and this will allow for more repetitions in sets until exhaustion [58]. NO_3_^−^ supplementation could also improve muscle efficiency and contractile capacity by promoting the release of calcium from the sarcoplasmic reticulum in the muscle cells and its reuptake [52, 69]. Thus, a train of action potentials leading to an increased supply of calcium to the muscle fibre will increase the strength of muscle contraction [13].

#### Effects of chronic supplementation with beetroot juice on intermittent high-intensity exercise efforts

Some sport modalities such as team, racket or combat sports require bursts of high-intensity efforts followed by rest periods. Thus, in team sports, high-intensity efforts (~3–4 s) are interspersed with variable active rest periods [70]. In racket sports like tennis, efforts last 7–10 s and rest periods 10–16 s (between points) and/or 60–90 s (side changes) [71]. Finally, in combat sports more intense efforts are 15–30 s long and active rest periods are 5–10 s long every 5 min [72]. In all these sports modalities, the capacity to repeat high-intensity efforts with only short recovery periods is considered a performance indicator [73]. This means that higher level athletes are able to maintain performance in successive high-intensity intervals over a long time period [74].

To find out if beetroot juice supplementation would improve this ability to repeat high-intensity efforts during a team sport match, Thompson et al. [56] administered beetroot juice over 7 days to a group of athletes (12.8 mmol NO_3_^−^). The performance test consisted of two blocks of five 6-s sets of sprints on a cycle ergometer with 14-s active recovery periods in the middle and end of a simulated match lasting 2 × 40 min [56]. The results of this study indicated a total work volume improved by 3.5% in the whole session, though this improvement was greater at the end of the first half (at half time).

If we again consider the nature of this type of exercise, it has been established that it involves the recruitment of type II muscle fibres [75, 76], which are more powerful though show more fatigue than type I units [77]. This lesser resistance to fatigue has been related to reduced blood flow and myoglobin concentrations in these muscle fibres compared to type I. Hence, type II muscle fibres are designed to promote non oxidative pathways and have shown a greater creatine storage capacity [78] for an enhanced metabolism of phosphocreatine [79] and proteins with a buffering effect at the intracellular level such as carnosine [80], favouring a glycolytic type metabolism.

Animal studies have shown that increased blood flow in response to NO_3_^−^ supplementation is greater in type II compared to type I muscle fibres [20]. This greater irrigation and oxygen availability in the recovery period along with a greater creatine storage capacity of motor type II units [78] (promoting phosphocreatine resynthesis [79]) means that during an exercise effort followed by a short rest period (14 s), beetroot juice supplementation could delay phosphocreatine depletion during successive sprints and explain the improvements noted by Thompson et al. [56].

Despite such greater effects of NO_3_^−^ supplementation on type II versus type I muscle fibres, animal studies have also shown that effects on calcium release and reuptake in the muscle cell sarcoplasmic reticulum is greater in type II than type I muscle fibres [52]. Accordingly, because of the important role of type II muscle fibres during sprints [75, 76], supplementation could have led to an improved capacity to generate muscle power and thus explain the significant improvements in performance observed by Thompson’s group.

Buck et al. [55] examined the effects of 6 days of supplementation with beetroot juice (6.4 mmol NO_3_^−^) or sodium phosphate (50 mg·kg lean mass) on performance in a test consisting of repeated sprints as 6 sets of 20 m and 25-s of rest between sets in the middle and end of a simulated match lasting 60 min. The beetroot juice intervention did not improve performance at these sprints, yet did do so when taken along with sodium phosphate (2%) compared with placebo, though this improvement was of lesser magnitude than when the subjects only took sodium phosphate supplements (5%). These findings suggest that, unlike beetroot juice, sodium phosphate intake may have an ergogenic effect in this protocol. If we compare the tests used by Buck et al. [55] and Thompson et al. [56], work periods were shorter (2–3 vs 6 s), while rest periods were longer (25 vs 14 s). Therefore it could be that 2–3 s efforts lead to a significantly lower reduction of phosphocreatine reserves at the end of these efforts. Further, the 25 s of rest approaching the 30 s in which the recovery of 50% of phosphocreatine stores takes place [67], may have been sufficient to stabilize reserves of phosphocreatine and therefore avoid the appearance of fatigue [81].

Another study investigated the effects of longer term supplementation (5 days) with beetroot juice (8.4 mmol NO_3_^−^), this time on performance in a repeated high-intensity test [60]. These authors sought to determine supplementation effects on different exercise protocols. Subjects performed a session consisting of twenty four 6-s sets of work and 24 s of rest between sets, a second session of two 30-s sets of work and 2 min of rest between sets and a third session of six 6-s sets and 60 s of rest between sets. As did Thompson et al. [56], Wylie et al. [60] selected 6-s exercise sets in the first session though rest intervals were longer (24 vs 14 s). Another difference was that the participants had not first undergone fatigue (in the simulated team sport match) before the performance test. Notwithstanding, results were similar in that mean power generated in the sets over a whole session improved by ~7%. However, improvements across the 24 × 6–24 protocol were not comparable to those recorded in the other two tests, in which no significant improvements were recorded.

In the test protocols including 30-s and 60-s work efforts, beetroot juice supplementation resulted in no improvements in any indicators of performance [60]. These protocols consisting of longer duration work intervals mainly involve a glycolytic type metabolism and in smaller measure elicit the high-energy phosphagen system. An increase in glycolysis leads to increased H^+^ production, lowering pH [82]. To avoid increasing acidosis, a series of responses targeted at reducing phosphofructokinase take place including diminished glycolysis [83] and phosphocreatine resynthesis [84], and muscle contractibility modifications [85]. Such responses manifest as reduced non aerobic metabolism or a reduced capacity for muscle power and strength, in other words, fatigue [86]. Supplements such as β-alanine (which increases muscle carnosine concentrations [87], a protein that acts as a buffer inside the cell [88]) and sodium bicarbonate [89] (main extracellular buffering agent) have shown ergogenic effects on performance at high-intensity efforts involving the predominance of glycolytic metabolism [90]. The combined effect of these supplements is greater than the impact of each supplement on its own [91].

Although beetroot juice supplementation induces vasodilation and increased blood flow (in type II muscle fibres, recruited mainly in exercise bouts of 30 to 60 s duration), increasing available oxygen in the muscles, rather than being activated because of a lack of oxygen (anaerobiosis), non-oxygen dependent pathways are activated because of a greater demand for energy production via oxidative phosphorylation. Thus, these effects, although they potentiate oxidative phosphorylation, have no repercussions on glycolytic energy metabolism. Hence, as beetroot juice has no alkalizing effect supplementation with this product is unable to reduce acidosis, as the main factor limiting performance at efforts lasting 30–60 s. However, potentiating effects on aerobic metabolism increases the speed of phosphocreatine resynthesis, dependent on oxidative phosphorylation. This means it may be effective for repeated high-intensity efforts whose duration is close to 6–10 s, in which high energy phosphagens contribute mainly to the metabolism [92] and the work volume is sufficient to cause significant depletion, which when faced with short rest intervals leads to progressive depletion and consequently to fatigue. Accordingly, beetroot juice supplements can have an ergogenic effect when exercise efforts are intermittent, maximum intensity, short-duration (6–10 s) and interspersed with brief recovery periods (<30 s).

### Effects of acute beetroot juice supplementation on intermittent high-intensity efforts

Five of the studies reviewed here were designed to analyze the effects of a single beetroot juice supplement on intermittent high-intensity exercise efforts [38, 53, 54, 57, 59]. Aucouturier et al. [54] administered the supplement (~10.9 mmol NO_3_^−^) to a group of recreation athletes 180 min before performing sets until exhaustion consisting of 15 s of pedalling at 170% VO_2max_ followed by 30-s rest periods. The authors reported that the beetroot supplement gave rise to improvements close to 20% in the number of repetitions performed and the total work completed in the session [54]. Besides the number of sets completed and the work accomplished, these authors measured red blood cell concentrations at the microvascular level. The beetroot juice, apart from improving performance, was found to increase microvascularization. Such improvements are considered a beneficial effect on oxygen exchange in the muscle [93]. Accordingly, these oxygen availability improvements produced at the muscular level could have potentiated oxidative phosphorylation during rest periods, and, given their brief duration, could have increased phosphocreatine resynthesis when subjects took the supplement rather than the placebo. Thus, supplementation would have delayed the depletion of phosphocreatine reserves and this effect was likely the cause of the improvements observed in the repeated sets of intermittent sprints [94, 95].

As did Aucouturier et al. [54], Muggeridge et al. [38] examined the effect of beetroot juice (5 mmol NO_3_^−^) taken 180 min before an intermittent effort consisting of 5 sets of 10 s in a kayak ergometer with 50-s interset rest periods. In this study, though supplementation seemed to have a greater effect on the power generated in the last two sets, the improvement noted lacked significance. However, if we compare this study with the study by Aucouturier et al. [54], work periods in the Muggeridge study [38] were shorter (10 vs 15 s) and rest periods were much longer (50 vs 30 s). Ten second maximum intensity intervals have a significantly reduced capacity compared with 15s intervals to deplete phosphocreatine reserves. Moreover, the rate of phosphocreatine replacement has a first phase in which up to 50% of these reserves can be replenished in 30 s and 100% in 3–5 min [67]. Also if we consider that the main effect of beetroot juice supplements is linked to an improved rate of phosphocreatine resynthesis, it is possible that as there is less depletion and a rest period in which there is almost complete recovery of phosphocreatine reserves, supplementation could not have exerted any beneficial effect in the study by Muggeridge et al. [38]. However, despite the short work periods and relatively long recovery periods and the fact that the power developed in the last sets showed an improved trend following supplementation, it is possible that lengthening intervals in a set until exhaustion would have been beneficial and given rise to similar results to those observed by Aucouturier et al. [54].

Rimer et al. [59] assessed the effects of acute supplementation (150 min before exercise) with beetroot juice (11.2 mmol NO_3_^−^) on performance in a maximal intensity 3-s test on an isoinertial cycle ergometer and a 30-s test on an isokinetic cycle ergometer. Supplementation was effective at improving pedalling cadence, and thus the power generated, in the 3-s test. However, no such effect was observed in the isokinetic test.

The improvements noted by Rimer’s group in the 3-s test affected pedalling cadence. Because of the link between such improvements and an increase in muscle shortening velocity [96] and the proposal that NO could increase this velocity [97, 98], the authors suggested that beetroot juice could have a beneficial effect on power output [59]. This rationale was also used to explain the lack of changes produced in the 30-s test in which pedalling cadence was fixed at 120 rpm. This means that any improved power production in the isokinetic test could only occur if there was an increase in power at a constant shortening velocity [59], since power equals force times velocity.

In a later investigation performed in CrossFit athletes, it was reported that supplementation with NO_3_^−^ salts (8 mmol NO_3_) rather than beetroot juice was able to improve performance in a 30-s cycle ergometry test [99]. However, unlike the 30-s test used by Rimer et al. [59], the test was isoinertial. The difference between the 2 cycle ergometers is that while in the isokinetic test pedalling cadence is prefixed and improvements only in strength are possible, in an isoinertial test the workload is fixed and any power improvements produced manifest as improvements in pedalling cadence. Given that beetroot juice supplementation could improve power development as a consequence of a reduced muscle shortening velocity [59, 97, 98], the isokinetic cycle ergometer is perhaps not sufficiently sensitive to assess the effects of this supplementation. Considering the beneficial effects on cadence and power output observed in the cycle ergometry 3-s [59] and 30-s [99] tests, it seems that beetroot juice supplementation could have a beneficial effect on this type of effort.

In a fourth study, Clifford et al. [57] assessed the effects of a single intake of beetroot juice on performance in a test of 20 sets of 30 m sprints interspersed with 30-s rest periods. These authors observed no ergogenic effects of the supplementation. However, if we look at the characteristics of the test employed by the researchers, we find that the work periods (close to 3 s) together with the 30 s recovery periods could be sufficient for the subjects to have recovered their phosphocreatine levels in the rest intervals, minimizing the possible ergogenic effects of the supplementation.

A novel indicator used in this study by Clifford et al. [57] was the counter-movement jump (CMJ) test performed before the intermittent velocity test and in the rest periods. Performance in this test is determined by the contractile properties of muscle and by neuromuscular control of the entire musculoskeletal system [100]. Given that fatigue reflects the incapacity of the neuromuscular system to maintain the level of power required [101], losses in CMJ height at the end of exercise are taken as an indicator of muscular fatigue [102].

In the study by Clifford’s group [57], it was observed that the protocol of intermittent sprints gave rise to muscular fatigue. This fatigue can be the outcome of deficiencies in the muscle’s contractile mechanism [101, 103]. Alternatively, strong eccentric actions of the hamstring muscles during sprints may produce muscle damage [104] and therefore modify the structure of the muscle fibre’s sarcomeres. Thus, any loss in CMJ height could indicate muscle damage. While CMJ was monitored after the protocol of 20 sets of 30 m with 30-s rest periods, a greater recovery of CMJ height was observed in the supplementation group. This suggests that beetroot juice could help preserve muscle structure during high-intensity efforts. Another explanation could be related to the vasodilation effect of beetroot juice [50] possibly helping muscle regeneration during early recovery. In future work, biomarkers of muscle damage or inflammation need to be examined.

In the fifth study, Martin et al. investigated the effects of beetroot juice (6.4 mmol NO_3_^−^) on repetitive sets until exhaustion each consisting of 8 s of work followed by 30 s of rest on a cycle ergometer [53]. No effects were detected on power output in the different sets. Moreover, a lower number of sets was accomplished in the session for the supplementation group versus placebo group. In effect, this was the only study to describe an ergolytic effect of beetroot juice. The authors argued that because of the scarce contribution of oxidative phosphorylation to energy metabolism during high-intensity efforts and that the ergogenic potential of this supplement is related to potentiating oxidative pathways, no beneficial effects are produced on this type of physical action.

The results of the investigation by Martin et al. [53] conflict with those of others who did observe beneficial effects on performance in similar tests [54, 56, 58, 60]. Beetroot juice was taken 120 min before exercise. This regimen is not appropriate, as peak NO_2_^−^ levels are produced 2–3 h after ingestion and it is recommended that supplementation should be taken at least 150 min–180 min before the high-intensity effort [32, 50]. Effectively, Aucouturier et al. [54] used a test of similar characteristics but the beetroot supplement was taken 180 min before the exercises, as recommended.

## Conclusions

To date, few studies have examined the effects of supplementation with beetroot juice on short-duration high-intensity exercise efforts [38, 53–60] and observations so far will need confirmation in future studies:

- Supplementation with beetroot juice has been shown to diminish the muscular fatigue associated with high-intensity exercise efforts, though it is not known if this is achieved by reducing fatigue and muscle damage and/or promoting muscle regeneration postexercise.
- When faced with exercise efforts that could considerably deplete phosphocreatine reserves (sets of resistance training or repetitive sprints of around 15 s interspersed with short rest periods) and given that phosphocreatine resynthesis requires an oxidative metabolism, beetroot juice could help the recovery of phosphocreatine reserves and thus avoid its depletion during repeated efforts. In parallel, supplementation would limit the build-up of metabolites such as ADP and inorganic phosphates, which are known to induce muscular fatigue.
- Beetroot juice has been shown to improve the release and reuptake of calcium at the sarcoplasmic reticulum. This could help the power production associated with improvements in muscle shortening velocity. Non-isokinetic ergometers (in which movement velocity is not assessed) are sensitive to such improvements in power generation.

### Study limitations

The main limitation of our review is the scarcity of studies that have examined the effects of beetroot juice supplementation on intermittent, high- intensity exercise. This limitation is also magnified by the varied design of the few studies available including different supplementation doses and regimens.

### Future lines of research

- As it has been proposed that beetroot juice supplementation improves phosphocreatine resynthesis during the brief rest periods included in protocols of intermittent high-intensity exercise, future studies are needed to confirm via a muscle biopsy phosphocreatine levels during repeated high-intensity efforts.
- To examine the possible beneficial effect of beetroot juice on muscle shortening velocity reflected as improved pedalling cadence, future studies need to assess the ergogenic effect of this supplement in a single, constant-load test on an inertial cycle ergometer.
- To elucidate the mechanism whereby beetroot juice diminishes muscular fatigue and improves recovery from this fatigue, the effects of ingesting NO_3_^−^ on biomarkers of inflammation and muscle damage need to be addressed.
- According to the results of the study in which an ergolytic effect was produced in response to a single dose of beetroot juice administered 120 min before exercise, future investigations should determine the most appropriate timing of supplementation to optimize its ergogenic potential.
- Finally, owing to the possible beneficial impacts of beetroot juice, we will need to assess the interactions of beetroot juice with other supplements of proven ergogenic effects in this type of exercise effort such as caffeine, creatine, β-alanine and sodium bicarbonate.
